# Application of personalized templates in minimally invasive management of coronal dens invaginatus: a report of two cases

**DOI:** 10.1186/s12903-024-04377-5

**Published:** 2024-05-22

**Authors:** Mingming Li, Guosong Wang, Fangzhi Zhu, Han Jiang, Yingming Yang, Ran Cheng, Tao Hu, Ru Zhang

**Affiliations:** 1 State Key Laboratory of Oral Diseases & National Center for Stomatology & National Clinical Research Center for Oral Diseases & Department of Preventive Dentistry, West China Hospital of Stomatology, Sichuan University, Chengdu, Sichuan China; 2 Department of Endodontics, School of Stomatology, Capital Medical University, Beijing, P. R. China

**Keywords:** Guided endodontics, Minimally invasive endodontics, Coronal dens invaginatus, Templates

## Abstract

**Background:**

Treating the coronal dens invaginatus (CDI) with pulp infection commonly involves the removal of invagination, which increases the risk of perforation and fracture, and compromises the tooth structure. Minimally invasive endodontic management of CDI is highly recommended. This report describes two cases of type II CDI with the application of personalized templates.

**Case presentation:**

Two cases of type II CDI, affecting the main root canal in a maxillary canine and a lateral incisor, were diagnosed. A guided endodontics (GE) approach was applied. Cone-beam computed tomography and intraoral scans were imported and aligned in a virtual planning software to design debridement routes and templates. The MICRO principle (which involves the aspects of Mechanical (M) debridement, Irrigation (I), Access cavities (C), Rectilinear routes (R), and Obstruction (O)) was proposed for designing optimal debridement routes for future applications. The templates were innovatively personalized and designed to preserve the tooth structure maximally while effectively debriding the root canal. Root canal treatment with supplementary disinfection was then performed. The follow-up of the two patients revealed favorable clinical and radiographic outcomes.

**Conclusions:**

The GE approach could be a feasible method for preserving healthy dental structure while effectively debriding the root canal, thereby achieving successful and minimally invasive endodontic treatment for CDI.

**Supplementary Information:**

The online version contains supplementary material available at 10.1186/s12903-024-04377-5.

## Background

Dens invaginatus (DI) is a developmental tooth anomaly caused by the invagination of the enamel organ into the dental papilla (coronal dens invaginatus, CDI) and/or the infolding of Hertwig’s root sheath into the root (radicular dens invaginatus, RDI) before mineralization occurs [1]. CDI is more common than RDI, with a prevalence ranging from 0.04% to 10% [2] and of 8.47% in China [3]. The maxillary lateral incisor is the most frequently affected tooth, while canines represent only 4.6% of all teeth affected by DI [4].

CDI has diverse clinical manifestations, encompassing multiple shapes and sizes [5]. The thin lining of the invagination can retain plaque and food debris, thereby increasing the risk of caries, pulp necrosis, periapical inflammation, and even odontogenic cyst formation [6]. Oehlers categorized CDI into three types based on the extent of invagination: type I (65.9%), type II (29.5%), and type III (4.6%) [2].

The treatment options for CDI vary based on the specific type of CDI and the condition of the pulp. Generally, the principle is to maintain the pulp vitality or preserve the tooth by the least invasive method [1]. Preventive filling is the proper approach for CDI with no pulp/periapical infection. With the use of cone beam computed tomography (CBCT), dental microscope, and ultrasonic instruments, CDI with pulp necrosis/periapical lesions, which formerly required extraction, could now be preserved to some extent by endodontic treatment and surgery when necessary [7, 8]. However, the complex and irregular anatomy of the canal system poses significant challenges to endodontic treatment, especially in cases of type II and type III CDI. Traditionally, endodontists have commonly removed the invagination and proceeded with root canal treatment (RCT) [9]. This approach heightens the risk of perforation or fracture, compromises tooth structure, and complicates the filling and restoration process, potentially leading to endodontic treatment failure [10, 11]. Therefore, it is essential to preserve the maximum amount of healthy structure and effectively debride the root canal.

Guided endodontics (GE) is a computer-guided technique that involves three-dimensional imaging via CBCT and digital surface scanning, both of which are integrated into planning software. Subsequently, virtual planning entails mapping the access cavity and creating a precise template to direct the bur towards the intended target point [12]. *In vitro* studies demonstrated acceptable accuracy in access cavity preparation using endodontic guides [13]. GE has been widely utilized in teeth with root canal calcification and periapical periodontitis, aiming for minimally invasive access cavity preparation and root canal location [12, 14]. However, the successful application of GE in teeth with CDI remains underreported, and there is currently no standardized principle for designing debridement routes due to the complex root canal systems in CDI.

This report describes the management of teeth with type II CDI using an improved GE approach under the MICRO principle. The goal is to advance CDI treatment and provide suggestions for managing CDI cases in clinical practice.

## Case presentation

### Case 1

**Fig. 1:**
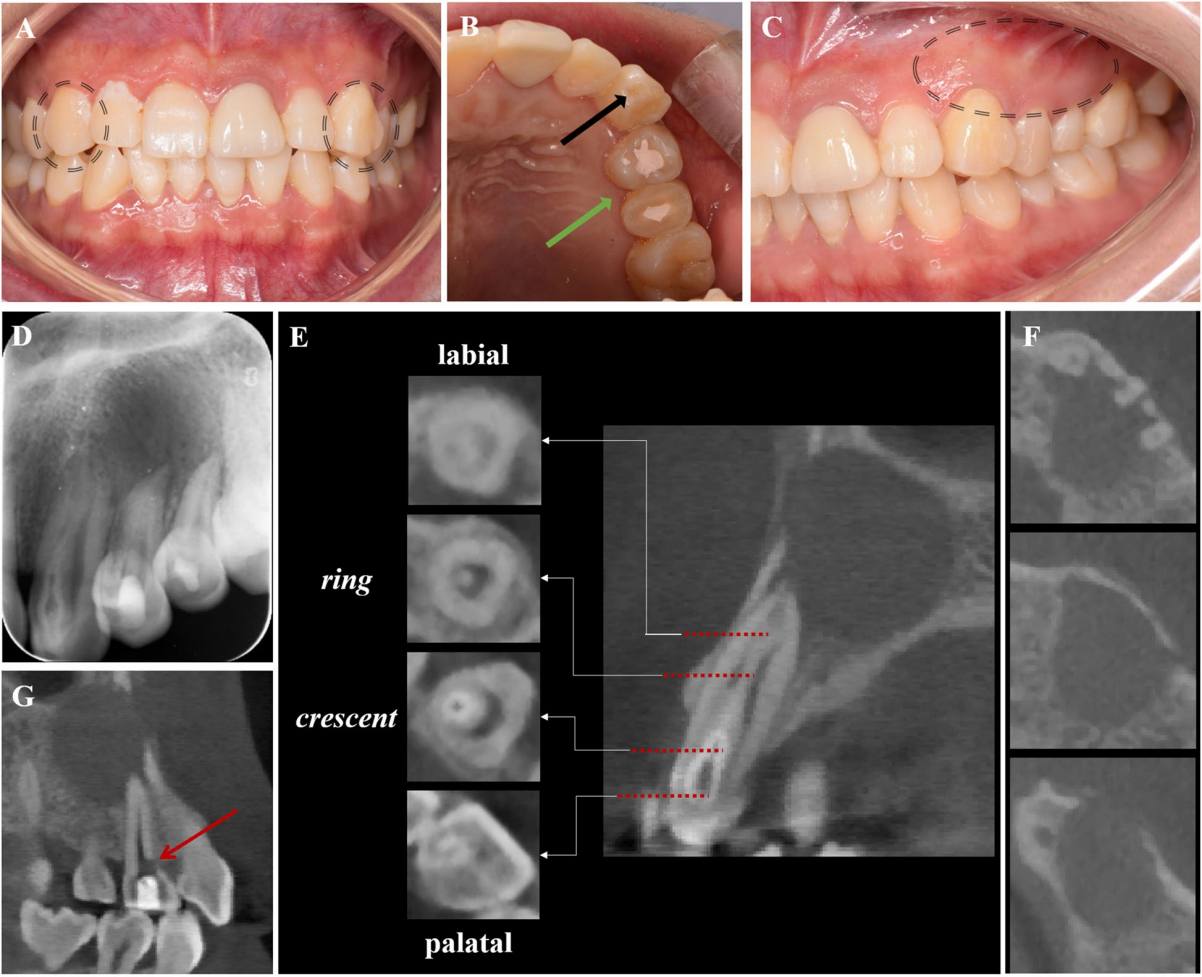
Preoperative examination of case 1. **A** Tooth 23 exhibited a larger mesiodistal diameter than tooth 13. **B** Green arrow: temporary sealing material on the occlusal surface of teeth 24 and 25; black arrow: a small foramen caecum on the mesial part of the palatal surface of tooth 23. **C** No swelling or sinus tract was observed at the buccal/labial alveolar mucosa related to teeth 23-25. **D** The periapical radiograph revealed CDI in tooth 23 and a radiolucent lesion in the periapical region of teeth 23-25. **E-G** CBCT scan images: **E** Axial slices at various points denoted on the sagittal section. The upper half of the invagination appeared tooth-shaped, compressing the true root canal into a crescent, while the lower half showed an irregularly cylindrical shape, compressing the true root canal into a ring. **F** The periapical radiolucency extended from the mesial side of tooth 23 to the distal side of tooth 25. **G** A lateral perforation on the mesial wall of tooth 24

**Fig 2:**
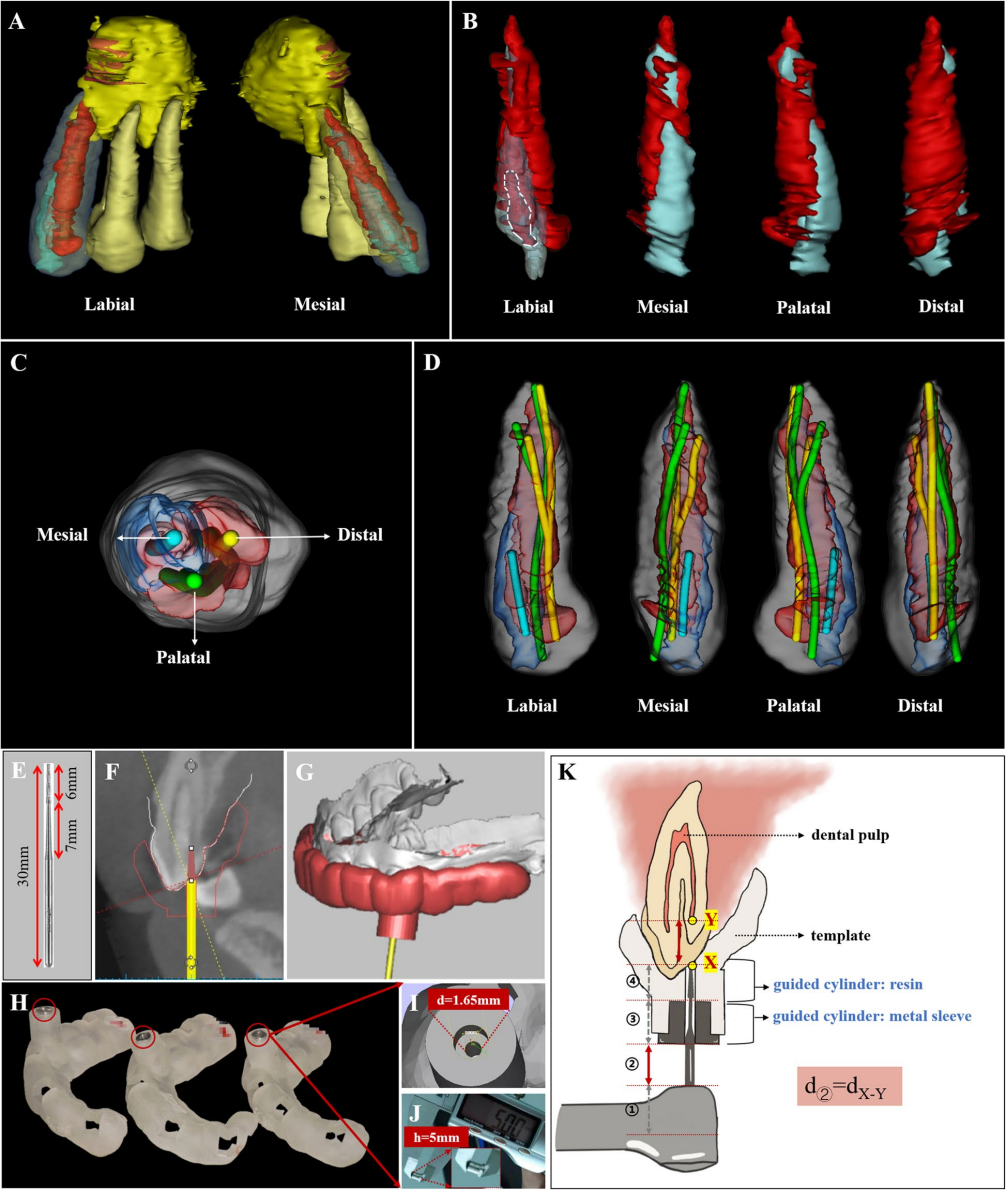
Virtual simulation of the debridement routes and the fabrication of the templates of case 1. **A** Three-dimensional reconstruction of teeth 23-25 and the periapical lesion. **B** Internal structure and distribution of the pulp of tooth 23 (red: pulp; blue: invaginated tooth; white line: pulp in the invaginated tooth). **C-D** Virtual simulation of three opening access cavity positions and rectilinear debridement routes for the root canals of tooth 23, designed according to the MICRO principle. **E** Information on the dental bur used. **F** A virtual copy of the bur. **G** Templates designed and matched to the maxillary arch and dental bur. **H** Three-dimensional printer fabricated three templates. **I, J** Information on the stainless-steel sleeve (d, diameter; h, height). **K**: Schematic diagram depicting the action of the dental bur, guided by personalized templates. “d_**②**_=d_X-Y_” indicates that “the distance of **②** is equal to the distance from X to Y”. When the bur is fully inserted into the cylinder, it reaches the pulp cavity

A 24-year-old woman was referred from a private dental clinic to the dental hospital due to recurrent swelling and pain in the vestibular region of teeth 23-25 despite receiving endodontic treatment over the past two weeks. Medical and family history were noncontributory. The extraoral examination showed no apparent abnormalities. The intraoral examination revealed temporary sealing materials on the occlusal surface of teeth 24 and 25 (Fig. 1B, green arrow). Compared with the contralateral canine, tooth 23 exhibited a larger mesiodistal diameter (Fig. 1A) and had a small pit on the mesial part of the palatal surface of tooth 23 (Fig. 1B, black arrow). For teeth 23-25, there was no evidence of caries, restorations, increased tooth mobility, or increased probing depth. Pulp sensibility tests indicated no response to cold/hot or electric pulp tests. Some discomfort was experienced on direct vertical percussion, and slight pain was felt upon palpation of the periapical area. The buccal/labial alveolar mucosa related to teeth 23-25 was normal, with no swelling or sinus tract (Fig. 1C). The periapical radiograph (Fig. 1D) revealed Oehlers’ Type II CDI in tooth 23 and a radiolucent lesion in the periapical region of teeth 23-25. To elucidate the morphology of tooth 23 and the extent of the periapical radiolucency [15, 16], a CBCT scan (Morita, Kyoto, Japan) was taken with a voxel size of 125 μm and exposure parameters of 90 kV, 5.0 mA and 17.5 s with the patient's consent. The CBCT scan revealed the following findings: 1) The upper half of the invagination was tooth-shaped and highly compressed the true root canal into a crescent shape, while the lower half was irregularly cylindrical and compressed the root canal into a ring. 2) The periapical radiolucency extended from the mesial side of tooth 23 to the distal side of tooth 25 and involved the buccal wall of the alveolar bone but was not connected to the maxillary sinus. 3) A lateral perforation was observed on the mesial wall of the pulp cavity of tooth 24 (Fig. 1E-G). Three-dimensional reconstruction images of the complex canal morphology of tooth 23 using Mimics 19.0 (Materialise, Belgium) were shown in Figure 2A-B.

The diagnoses were type II CDI with apical periodontitis of tooth 23, apical periodontitis of teeth 24 and 25, and a suspected periapical cyst of left maxillary.

CBCT scans and stereolithography (STL) files of the maxillary arch obtained from an intraoral scanner (Trios T12P, 3Shape, Copenhagen, Denmark) were imported into virtual planning software (SimPlant, Materialise Dental, Belgium). After aligning both scans by the software, a virtual copy of the bur (Ref.: HF115-010-FXXL, Oko Dent, Tautenhain, Germany; Fig. 2E), with diameters of 1.0 mm on the working tip and 1.6 mm on the petiole part, was superimposed with its tip touching the top of the pulp cavity (Fig. 2F). The correct position of each virtual bur was verified in three dimensions. By analyzing the internal structure and pulp distribution of tooth 23, three optimal access cavities and debridement routes were virtually simulated to facilitate feasible and comprehensive removal of the infected pulp tissue. The virtual simulation was diagrammatically represented in Figure 2C-D. Subsequently, templates were designed to match the maxillary arch and dental bur (Fig. 2G). Each template had a guided cylinder of resin and a stainless-steel sleeve (Ling Zhiqi Precision Tools Technology Co. Ltd., Shanghai, China) to guide the dental bur into the pulp cavity. The height and diameter of the stainless-steel sleeve were 5 mm and 1.65 mm, respectively (Fig. 2I, J). The height of the resin sleeves was adjustable according to the working depth of the dental bur at each position. After that, the three templates were fabricated using a three-dimensional printer (ProJet MJP 3600, 3D Systems, South Carolina, America; Fig. 2H).

**Fig 3:**
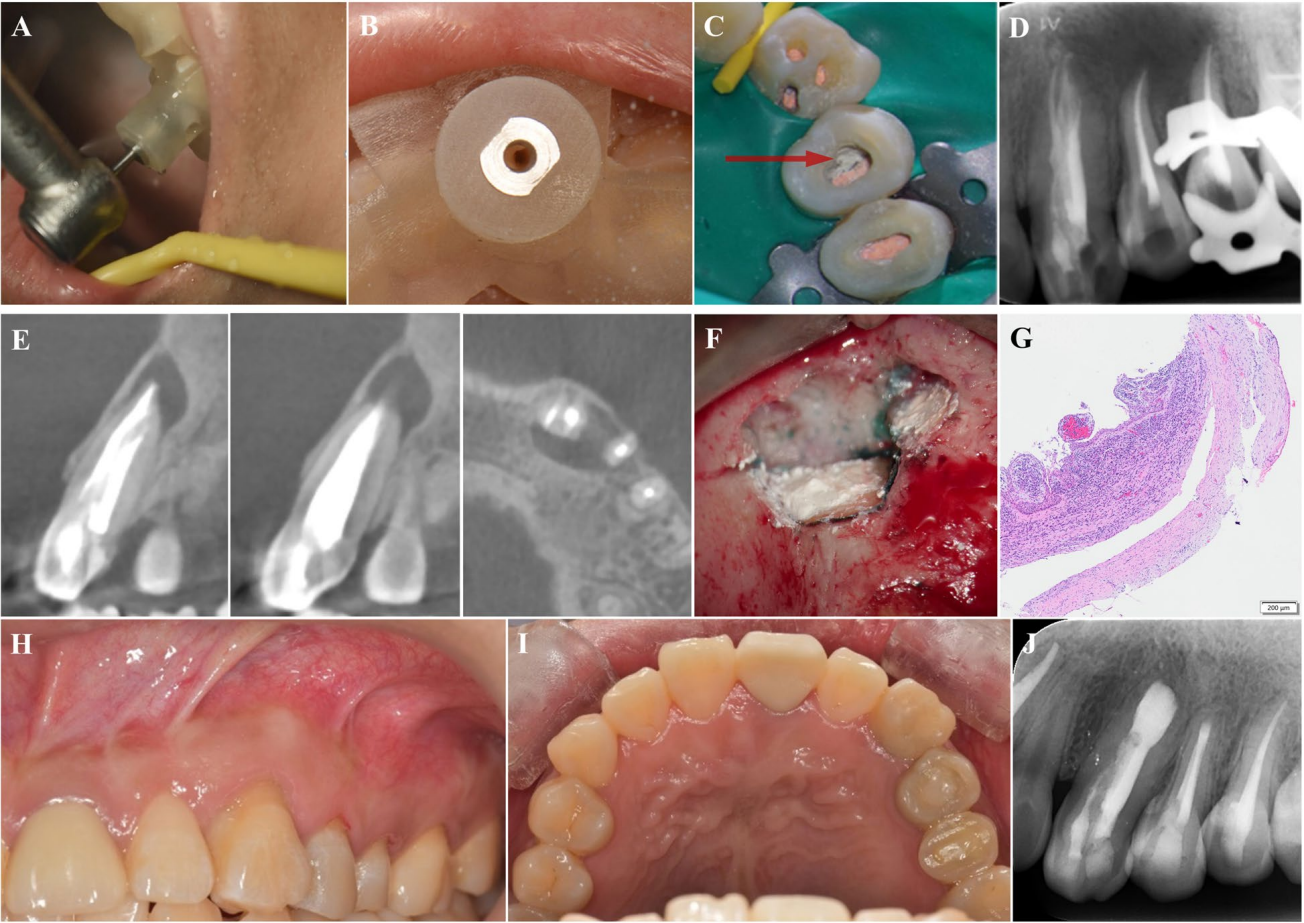
Treatment process and follow-up of case 1. **A**, **B** Opening of the pulp cavity guided by templates. **C**, **D** After root canal filling and verification, the perforation of tooth 24 was repaired with MTA (red arrow). **E** A CBCT scan taken 14 months later indicated good root canal filling and reduced lesion size. **F** Endodontic microsurgery. **G** Histopathological examination. **H**-**J** Follow-up: **H**, **I** Intraoral examination; **J** Radiographic images taken 12 months after endodontic microsurgery

Next, the correct fit was checked. When the bur was fully inserted into the cylinder, it reached the pulp cavity. Three minimally invasive access cavities were obtained using the templates (Fig. 3A, B). RCT of teeth 23-25 was performed. The working lengths were determined by an electronic apex locator (ProPex II, Dentsply, New York, USA). The root canals were prepared using an endodontic rotary instrumentation system (S3, SANI, Chengdu, China) to 30/.04 files and irrigated using an ultrasonic tip (ACTEON, France) with 3% sodium hypochlorite, 17% EDTA and 2% chlorhexidine. After the root canals were dried, calcium hydroxide dressing (Apexit Plus, Ivoclar Vivadent, Liechtenstein) was applied, followed by temporary filling (Caviton, GC, Japan). After 2 weeks, the root canals were sealed with a warm gutta-percha system (SuperEndo, BL, Korea) and a root canal sealer (iRoot SP, Innovative Bioceramix, Canada; Fig. 3C, D). The perforation of tooth 24 was repaired with MTA. Finally, the cavities were filled with resin (Z350 XT, 3M ESPE, Saint Paul, America).

Fourteen months later, the patient experienced a recurrence of mucosal swelling and pain. Slight pain was felt upon palpation of the periapical area. CBCT revealed that the periapical radiolucency extended from the mesial side of tooth 23 to the distal side of tooth 24, indicating a relapse of periapical infection (Fig. 3E). Then, endodontic microsurgery was implemented (Fig. 3F and Fig. S1). Histopathological examination revealed an inflammatory odontogenic cyst (Fig. 3G). A year of follow-up endodontic microsurgery revealed significant healing of the periapical lesion. The treated teeth were asymptomatic (Fig. 3H-J and Fig. S2).

### Case 2

**Fig 4:**
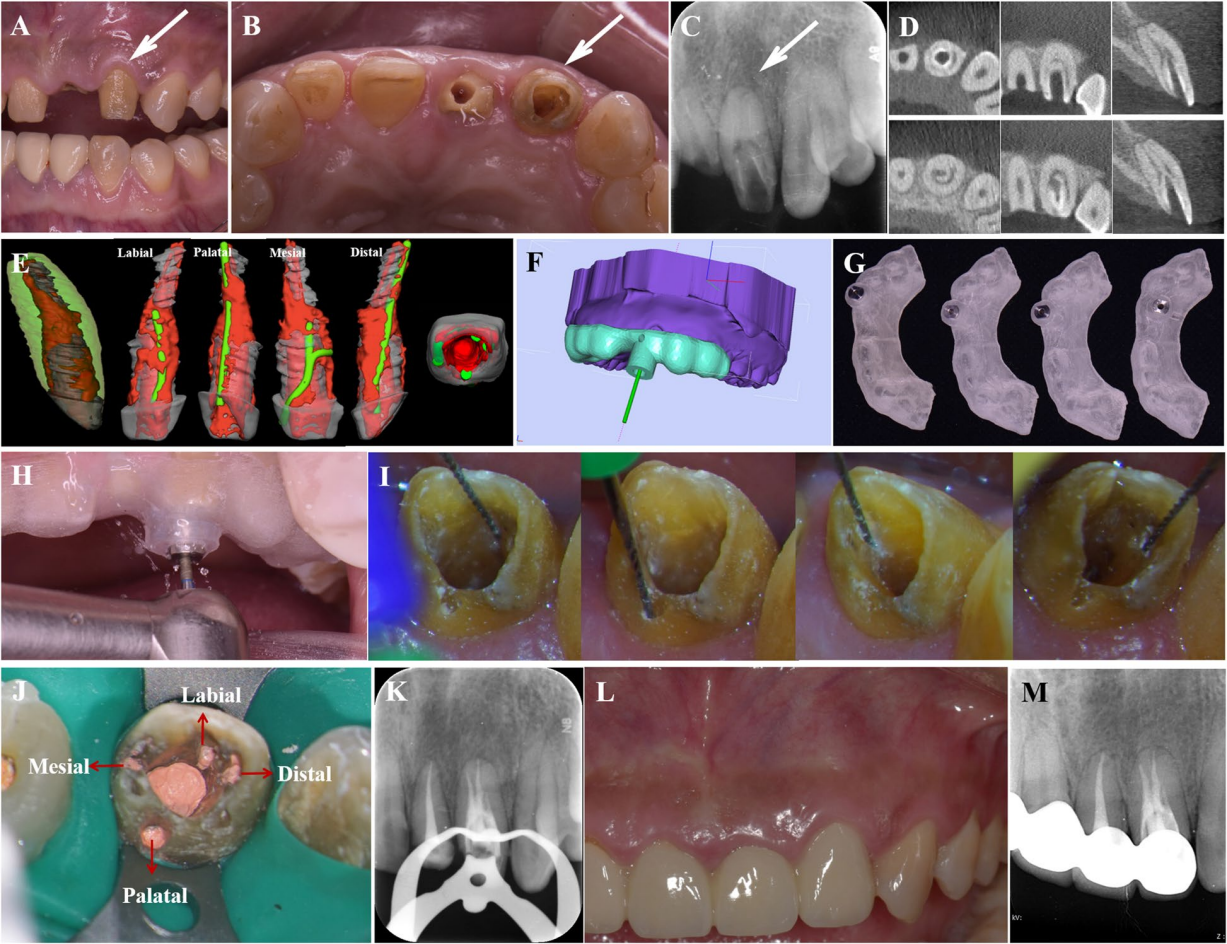
Treatment process and follow-up of Case 2. **A**, **B** Preoperative intraoral examination. **C**, **D** Preoperative periapical radiograph and CBCT scans. **E** Three-dimensional reconstruction of tooth 22 and virtual simulation of four debridement routes designed based on the MICRO principle. **F**, **G** Procedure for fabricating the templates. **H**, **I** Four opening access cavities guided by templates. **J**, **K** After root canal filling, the effect was verified. **L**, **M** 3-year follow-up

A 39-year-old woman was referred to the hospital due to pain in the left upper anterior tooth after crown preparation to correct tooth discoloration. Medical and family history were non-contributory. The extraoral examination revealed no apparent abnormalities, and the intraoral examination revealed crown preparation of tooth 22, along with an access cavity in the middle of its palatal surface (Fig. 4A, B, white arrow). There was no tooth mobility or increased probing depth. Pulp sensibility tests indicated no response to cold/hot or electric pulp tests. The tooth exhibited intense discomfort on direct vertical percussion and pain upon palpation of the periapical area. Radiographic examination revealed Oehlers’ Type II CDI in tooth 22. The upper half of the invagination was tooth-shaped and highly compressed the true root canal into a ring, while the lower half was irregularly cylindrical and compressed the root canal into a crescent shape. The periodontal ligament space was widened (Fig. 4C, D). Three-dimensional reconstruction images depicted the complex canal morphology of tooth 22 (Fig. 4E). The diagnosis was Type II CDI with acute apical periodontitis. Four debridement routes were designed, and corresponding templates were fabricated (Fig. 4F, G). GE-based RCT was implemented using the same procedure as described in case 1 (bur: TF-11, MANI, Utsunomiya, Japan; Fig. 4H-K). The three-year follow-up showed no obvious abnormalities, and the treated tooth was asymptomatic (Fig. 4L, M and Fig. S2).

## Discussion and conclusions

The occurrence of CDI in canines, particularly CDI with odontogenic cysts in maxillary canines, is exceedingly rare. Nevertheless, it is crucial to acknowledge the overall incidence of CDI, regardless of the position of affected tooth, is not low [17]. In Case 1, we speculated that the infection and cyst were the result of sustained inflammation originating from the infection of tooth 23 through the foramen caecum on its occlusal surface, which serves as the entrance to the invagination. Early identification and timely prophylactic treatment can prevent inflammatory sequelae related to CDI [18].

When the pulp of CDI is infected, GE-based RCT has emerged as a good approach for nonsurgical and minimally invasive treatment. Minimally invasive endodontics aims to remove the least amount of tooth structure while ensuring disinfection of the root canal system [19]. However, it is challenging to locate the access cavity precisely, achieve minimally invasive access cavities, and effectively eliminate infections due to the random variation and unique characteristics of each CDI-affected tooth. GE provides a solution by enabling endodontists to design templates and manage CDI with minimal tooth structure removal [20]. Few studies have demonstrated the positive impact of GE-based RCT on disease control (Table 1, [21–25]). However, a standardized protocol for designing optimal debridement routes using the GE approach in CDI cases is still lacking.

**Table 1:** Case reports on the use of guided endodontics for coronal dens invaginatus

**Year**	**Author**	**Affected tooth**	**Oehlers type**	**Periapical lesion**	**Endodontic surgery**	**Follow-up** **(Month)**	**Periapical outcomes**
2015 [21]	Álvaro Zubizarreta- Macho	22	II	+	—	6	healed
2017 [22]	Jesús Mena- Álvarez	21	I	+	—	12	healing
2019 [23]	Álvaro Zubizarreta- Macho	22	II	+	+	18	healed
2019 [24]	Afzal Ali	12, 22	II	+	—	12	healing
2019 [25]	Afzal Ali	12, 22	II	—	—	Not mentioned	Not mentioned

Virtual simulation was applied to visualize the distribution and direction of the debridement routes (Figs. 2C, D, and 4E; Fig. S3). The fundamental principle of debridement route design for CDI is to achieve maximal effectiveness with minimal tissue damage. This principle could be illuminated as the MICRO principle, which comprises the following key aspects: 1) Maximum mechanical (M) debridement. 2) Maximum chemical irrigation (I). 3) Optimal and accurate access cavity (C). 4) The most convenient rectilinear (R) route. 5) Minimal obstruction (O). In alignment with these aspects, the number of opening access cavities should be appropriate, the location should be accurate, and the interval should be reasonable. Well-designed debridement routes allow the instruments to enter rectilinearly, avoid obstruction, and maximize contact between the instruments and infected pulp tissue for efficient mechanical debridement and chemical irrigation.

Inspired by the 3-Determination principle, which involves determining the location, depth, and instrumentation angle in the management of calcified root canals [26], a personalized improvement was made to the templates compared to several CDI cases using the GE technique (Table 1). Besides verifying valid access location and direction, the ideal operating depth was also achieved by adjusting the height of the resin sleeve in the templates (Fig. 2K). When the bur was fully inserted into the cylinder, it reached the pulp cavity. This enhancement is advantageous, particularly for less experienced endodontists, as it reduces hesitation during the operation and allows for maximum dental structure preservation.

Given the complexity of the root canal system in CDI, enhanced supplementary disinfection was necessary. Chemical cleaning was intensified by ultrasonic irrigation with 3% NaOCl at 37°C, 17% EDTA, and 2% chlorhexidine [27].

CDI commonly occurs concomitantly with apical cysts. Currently, reliable guidelines regarding the use of nonsurgical or surgical approaches for treating periapical cysts still need to be developed [28, 29]. In case 1, nonsurgical RCT was administered initially, and the CBCT images revealed a significant reduction in the extent of the lesion (Fig. 3E). It is advisable to prioritize nonsurgical procedures. With proper infection control during the GE-based RCT procedure, the periapical lesion can heal or reduce in size, resulting in less trauma and shorter healing times when surgery is necessary [30, 31].

In summary, the GE approach could be a promising method for preserving healthy dental structure while effectively debriding the root canal, thus achieving successful and minimally invasive endodontic treatment for CDI. The MICRO principle is recommended for designing debridement routes in GE-based RCT. Additionally, personalized templates are recommended for easy access to the pulp cavity with precise depth control. These combined strategies offer promising prospects for enhanced treatment outcomes in CDI cases.

### Supplementary Information

Supplementary Material 1.

Supplementary Material 2.

## Data Availability

Data is provided within the manuscript and supplementary information files.

## Abbreviations

CDI: coronal dens invaginatus
GE: guided endodontic
CBCT: cone beam computed tomography
EDTA: Ethylenediaminetetraacetic acid
NaOCl: Sodium hypochlorite solution
