# Supplementary material for: Application of personalized templates in minimally invasive management of coronal dens invaginatus: a report of two cases
Source: BMC Oral Health. 2024 May 22;24:592. doi: 10.1186/s12903-024-04377-5 (PMC11110288; doi:10.1186/s12903-024-04377-5)
Supplement: Supplementary file 2 — Supplementary Material 2. [file 12903_2024_4377_MOESM2_ESM.docx]

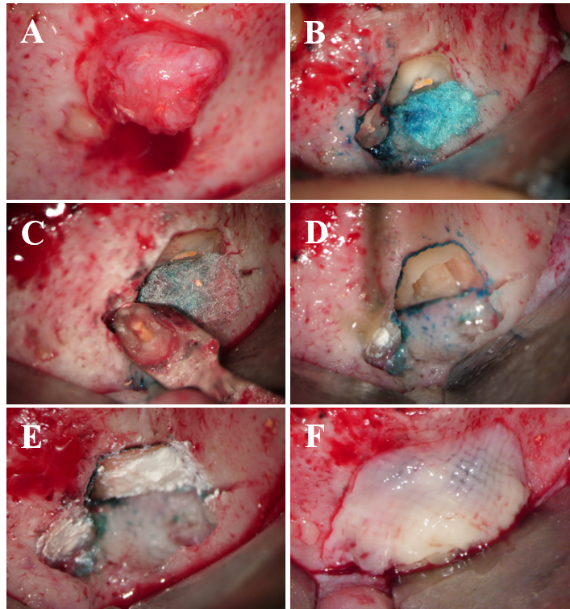


**Supplementary Figure 1. Treatment procedure of endodontic microsurgery.**

A. After administration of local anesthesia using articaine, a labial full thickness triangular mucoperiosteal flap was elevated to expose the pathological area. The granulomatous soft tissue around the root apex was curetted.

B. The root ends of 23-34 were resected 3 mm from the apex using a fissure bur.

C-D. A retrograde cavity of tooth 23 and 24 was prepared to a depth of 3 mm coaxially using ultrasonic tips.

E. The root-end cavities were refilled with iRoot-BP (Innovative Bioceramix, Canada).

F. Filled the autologous concentrate growth factors into the defect area, then covered it with a bio-membrane (Bio-Guide, Geistlich), and finally sutured the wound.


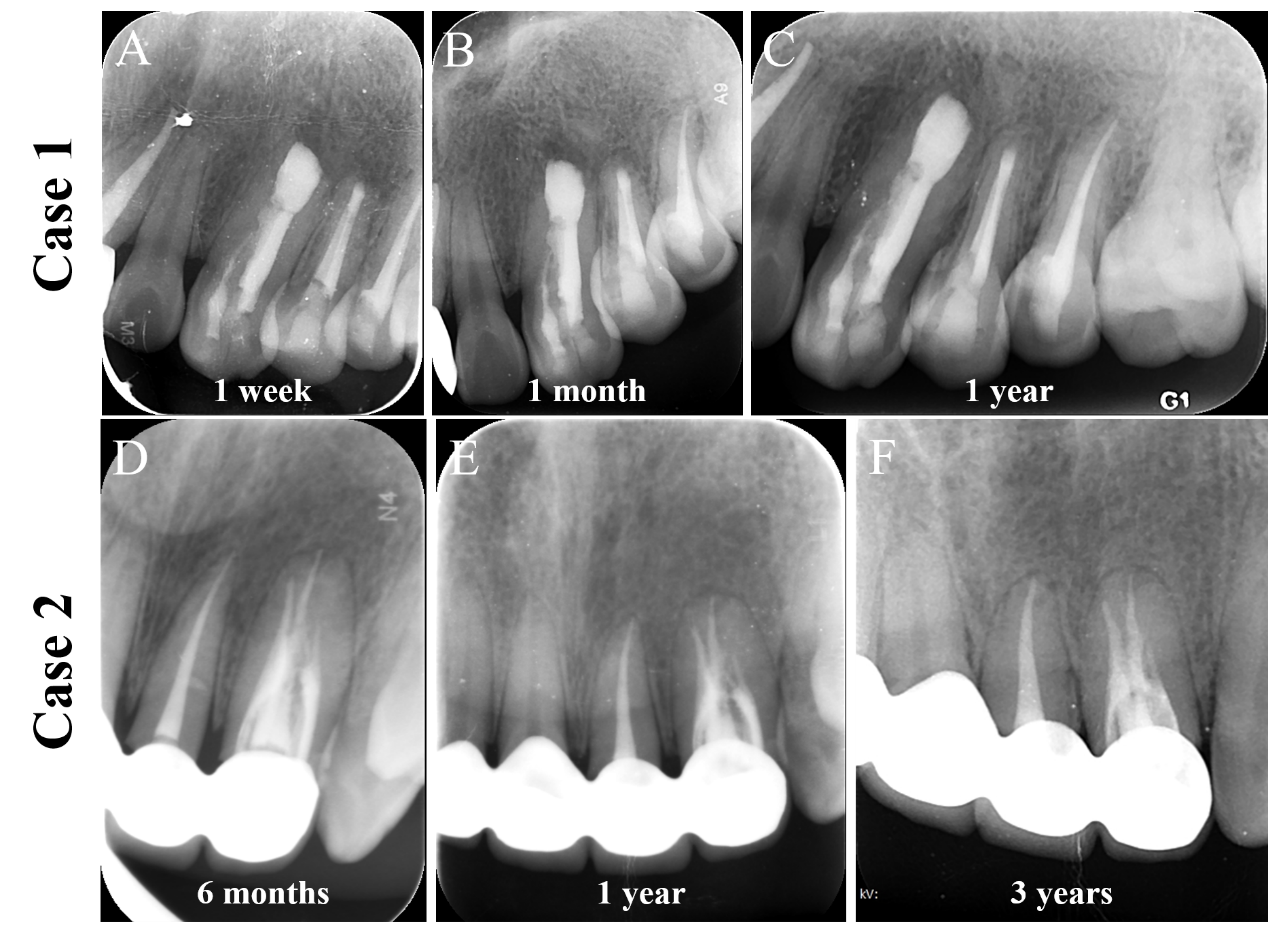


**Supplementary Figure 2: The follow-up of case 1 (following endodontic microsurgery) and case 2 (following GE-based RCT)**


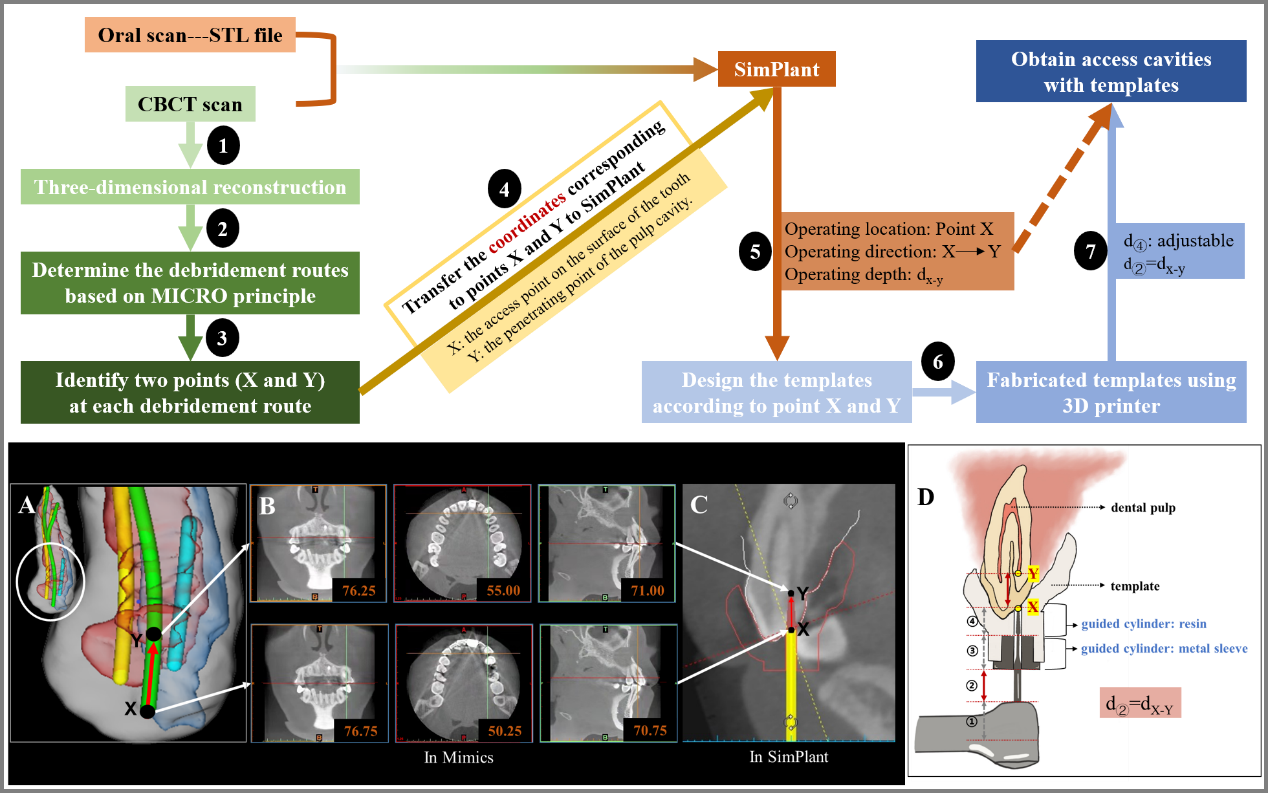


**Supplementary Figure 3. The process of designing and fabricating the personalized templates prior to RCT.**

1) Three-dimensional reconstruction based on CBCT scans: to master internal morphology and pulp distribution of tooth 23.

2) Debridement routes design based on three-dimensional reconstruction and MICRO principle: to facilitate feasible and comprehensive removal of the infected pulp tissue.

3) For each debridement route, we identified two points (Figure S3-A, B): Point X was the access point on the surface of the tooth, point Y was the penetrating point of the pulp cavity. Each point had a coordinate value.

4) The same points X and Y were identified in SimPlant through the coordinate values.

5) The templates were designed according to the points X and Y (Figure S3-C): a. The direction of the connection of points X and Y was the operating direction. b. The distance of the connection of points X and Y was the operating depth.

6) Fabricate the templates using a 3D printer.

7) Obtain accurate access cavities (with accurate location, direction and depth) under the guide of templates (Figure S3-D).
